# The Effect of Three Forms of Local Corticosteroids on Sinonasal Polyposis 

**DOI:** 10.22038/IJORL.2023.75247.3524

**Published:** 2024-01

**Authors:** Matin Ghazizadeh, Ali Goljanian Tabrizi, Najmeh Rajabi

**Affiliations:** 1 *Department of Otorhinolaryngology, Head and Neck Surgery, Taleghani Hospital, Shahid Beheshti University of Medical Sciences, Tehran, Iran.*

**Keywords:** Corticosteroids, Paranasal Sinuses, Recurrence

## Abstract

**Introduction:**

Since sinonasal polyposis (SNP) has a high recurrence rate after surgery, various studies have investigated the effect of corticosteroid medications to prevent disease recurrence. The present study was designed to compare the effect of three forms of local corticosteroids on preventing SNP recurrence post-operatively.

**Materials and Methods:**

This double-blind, randomized clinical trial study was conducted on 108 patients with SNP who underwent functional endoscopic sinus surgery (FESS). Permuted Block Randomization randomly assigned patients into three groups of 36 people: budesonide spray, betamethasone drop, and budesonide nebulizing suspension groups. One and six months after surgery, the patients were evaluated for recurrence of SNP by nasal endoscopy. SNOT 22 questionnaire was used to assess patients’ subjective improvement rate pre- and post-operatively.

**Results:**

According to the scores obtained in the Modified Lund-Kennedy Scoring, budesonide nebulizing suspension showed better effects on preventing the recurrence of sino-nasal polyps after FESS compared with betamethasone nasal drops. The score was significantly lower in the budesonide nebulizing suspension group compared to the betamethasone drop group (P=0.043). There was no statistically significant difference in the scores between the budesonide nebulizing suspension group and the betamethasone spray group (P=0.178). Also, we observed significant improvement in facial fullness in patients who received Budesonide nebulizing suspension.

**Conclusions:**

Budesonide nebulizing suspension, compared to betamethasone nasal drops, showed better effects on preventing the recurrence of SNP after FESS.

## Introduction

The precise underlying mechanism in the pathogenesis of sinonasal polyposis (SNP) is unknown, but it has been proposed that mucosal inflammation of the sinonasal cavities plays a crucial role in its pathogenesis (1,2). SNP can occur at any age, but it is more common in young and middle-aged people (3). Although surgery is not the first step in treating SNPs, it is indicated in patients with severe symptoms of nasal congestion, runny nose, and loss of taste, as well as in patients for whom medical treatment has not been effective (4). 

Functional endoscopic sinus surgery (FESS) is a standard, less complicated, and successful approach to treating sinonasal polyposis (5). However, the probable complications of FESS are bleeding, cerebrospinal fluid leakage, orbital complications, epiphora, obstruction of the sinuses orifice, recurrence, and some others (6). Various studies have shown that medical treatment along with surgery will be more effective for the treatment of SNP, and among drugs, perioperative corticosteroids cause fewer surgical complications and recurrence (1,7). 

Various studies have investigated the effect of corticosteroids on preventing SNP recurrence. Local corticosteroids are preferred due to minimal systemic side effects and easy usage. After FESS, the sinus cavities are opened, exposing the mucosa to local medications. The most effective forms of corticosteroids (drops, spray, suspension) should be determined to better SNP management. 

The present study is designed to compare the effect of three more accessible forms of local steroids (budesonide nasal spray, betamethasone nasal drops, and budesonide nebulizing suspension) on preventing the recurrence of SNP who underwent FESS. 

## Materials and Methods

The present randomized clinical trial was conducted between February 2020 and February 2022 in patients who underwent FESS. The study was approved by the Ethical Committee (IR.SBMU.MSP.REC.1399.782). Also, the Iranian Registry of Clinical Trial Identification code was IRCTID: IRCT20201021049095N1. 

All necessary issues, especially the possible side effects of drugs, were explained, and then the volunteer patients signed the informed consent form and entered the study based on the inclusion criteria. Healthy patients older than 18 who were involved with massive bilateral SNP and were candidates for a complete FESS (opening all paranasal sinuses) entered the study. The patients should not have any history of recent therapy with a systemic corticosteroid, previous sinus surgery, and underlying diseases, including cystic fibrosis, Kartagner, sarcoidosis, and vasculitis. The exclusion criteria include incomplete post-operative follow-ups and histological diagnoses other than SNP (such as a malignancy). 

The demographic information of the patients was registered. History of smoking and involvement with asthma and aspirin hypersensitivity (Samter's Triad) was determined. All patients underwent diagnostic endoscopy of the nose and paranasal sinuses to confirm the diagnosis. The Sino-Nasal Outcomes Test 22 (SNOT 22) questionnaire assessed patients’ subjective improvement rate pre- and post-operatively (6 months later). The Lund-Mackay staging system scores were determined by evaluating the paranasal sinus computed tomography (CT) scans(8). 

All patients were treated with the same medical methods post-operatively, using 20 ccs of normal saline wash four times daily. Local steroids were used every 12 hours after nasal rinsing. Participants were randomly divided into the following three groups by permuted block randomization method:

1- Budesonide (64 mcg/puff) nasal spray group (Koushan Pharmed, Tehran, Iran): The nasal spray prescribed two puffs on each nasal side, twice daily.

2- Budesonide (0.25 mg/ml) nebulizing suspension group (Koushan Pharmed, Tehran, Iran); Each unit should be diluted in 100 ml with %0.9 saline and pushed into each side of the nose using a 50-ml syringe twice daily. 

3-Betamethasone (% 0.1) nasal drop group (Darou pakhsh, Karaj, Iran); 2 drops were applied in both nostrils (4 drops in both) twice daily.Regular sino-nasal endoscopy was done monthly. If it was necessary to use systemic corticosteroids in the post-op period, it should be recorded. Lund-Kennedy endoscopic scoring system was determined after the sixth month to evaluate SNP recurrence(9). Also, all patients were assessed, considering possible drug side effects. The revision rate in each group was determined, too. Statistical analysis was done using SPSS version 22 statistical software (SPSS Inc., Chicago, IL, USA). First, the data distribution was evaluated using the Shapiro-Wilk statistical test. ANOVA statistical test was performed to compare the means in three groups before and six months after the study, and the Bonferroni approach was applied to compare each pair groups. A P <0.05 was considered statistically meaningful. 

## Results

The mean age of the participants was 48.58 ± 10.4 years, and 51% were female. Demographic and clinical characteristics are summarized in Table 1. 

Three groups were comparable according to the baseline variables. There was no significant statistical difference among the three groups considering comorbidities and underlying diseases.

**Table 1:** Characteristics of the study groups

Group	Budesonide nasal spray (n=36)	Betamethasone nasal drop (n=36)	Budesonide nebulizing suspension (n=36)	Total (n=108)
Age; Mean ± SD	47 ± 10.4	48 ± 9.3	50 ± 11.3	48 ± 10.4
Female; n (%)	16 (44)	20 (56)	19 (68)	55 (51)
Smoker; n (%)	10 (33)	9 (30)	11 (37)	30 (51)
Samter’s Triad	6 (17)	4 (11)	5 (14)	15 (14)
Comorbidities; n (%)				
Diabetes	6 (32)	7 (37)	6 (32)	19 (18)
Hypertension	11 (41)	11 (41)	5 (19)	27 (25)
Cardiovascular disease	2 (14)	6 (43)	6 (43)	14 (13)
Dyslipidemia	3 (43)	2 (29)	2 (29)	7 (7)
Chronic Kidney Disease	0 (5)	1 (33)	2 (64)	3 (3)

The recurrence of sinonasal polyposis according to Modified Lund-Kennedy Scoring six months after the surgery is compared and is given in Table 2. Analyses revealed a statistical significance in the recurrence score between the betamethasone nasal drop group and the budesonide nebulizing suspension group six months after the surgery (P-value = 0.043). Six months after surgery, it was 3.56 ± 1.64 in the betamethasone nasal drop group and 2.61 ± 1.59 in the budesonide nebulizing suspension group. There is no statistical significance in the effect of the betamethasone nasal drop compared to the budesonide nasal spray six months after the surgery (P-value = 0.999). Also, we found no significant difference in the effect of budesonide nebulizing suspension compared to the budesonide nasal spray six months after the surgery (P-value = 0.178).

**Table 2:** The recurrence of sino-nasal polyposis according to Modified Lund-Kennedy Scoring six months after the surgery in study groups

**group**	**Budesonide nasal spray (n=36)**	**Betamethasone nasal drop (n=36)**	**Budesonide nebulizing suspension (n=36)**	**P-value**
Before surgery (Lund Mackay Score)	20.61 ±2.71	19.89± 2.16	20.11± 2.42	0.441
Six months after surgery (Modified Lund-Kennedy Scoring)	3.33± 1.58	3.56± 1.64	2.61± 1.59	0.999^a^ 0.178^b^ 0.043^c^

a; Budesonide nasal spray group compared to Betamethasone nasal drop group

b; Budesonide nasal spray group compared to Budesonide nebulizing suspension group

c; Betamethasone nasal drop group compared to Budesonide nebulizing suspension group

Statistical analysis of the questionnaire revealed facial fullness was decreased significantly in patients who received budesonide nebulizing suspension (p value= 0.044). We did not find a significant difference in the severity of other symptoms.

The total number of patients and times of systemic corticosteroid usage among the three groups revealed no significant difference (P = 0.534). Six months after the operation, one patient from the budesonide nasal spray group (2.8%) and another from the betamethasone drop group (2.8%) underwent revision FESS. There was no revision in the budesonide nebulizing suspension group (P = 0.615).

The patients reported epistaxis and dry nose among the most common side effects of nasal corticosteroids. Epistaxis was not seen in the betamethasone drop group, but one patient from each of the budesonide nebulizing suspension and budesonide nasal spray groups (2.8%) was involved with recurrent self-limited epistaxis. Nasal dryness was observed in 2 cases (5.6%) in the betamethasone drop group, one patient (2.8%) in the budesonide nasal spray group, and nobody in the nebulizing budesonide suspension group. 

## Discussion

According to the current study, budesonide nebulizing suspension showed better effects on preventing the recurrence of sino-nasal polyps after FESS than betamethasone nasal drops. Although budesonide nebulizing suspension was more effective than nasal spray, the difference was insignificant. This observation may be due to a small sample size. On the other hand, betamethasone nasal drops and budesonide nasal spray did not differ significantly. So, the most efficient form of these three corticosteroids is budesonide nebulizing suspension, budesonide nasal spray, and betamethasone nasal drops in decreasing order. However, another study with a larger sample size must confirm the difference between budesonide nebulizing suspension and nasal spray.

Comparing the severity of patients' symptoms before and after the operation among the three groups revealed that the budesonide nebulizing suspension group showed significant improvement in the severity of the patient’s facial fullness, significantly affecting the patient’s quality of life. The amount of the efficient therapeutic substance of corticosteroids in all three groups is roughly equal. So, the significant improvement of these symptoms in the budesonide nebulizing suspension group may be due to a large amount of solution (100 ml o %0.9 saline) and the larger area of sinonasal mucosa exposed to local steroids. 

In a study conducted by Rawal and his colleagues(10), they randomly divided 50 patients with sino-nasal polyposis into two groups of 25: intervention (budesonide suspension user) and control (%0.9 saline user). Their results showed that budesonide suspension improved the patient’s quality of life based on the SNOT-22 questionnaire. It also improved olfactory function in these patients. 

The results of the present study also indicated that budesonide suspension was more effective in decreasing the patients' bothersome symptoms compared with budesonide nasal spray and betamethasone drop. In another study conducted by Wang et al. (11), the effect of nebulizing budesonide was investigated in patients with SNPs. 

Their study showed that nebulizing budesonide reduced the size of polyps, decreased the expression of interleukin 5, and increased the expression of tumor growth factor-beta and interleukin 10 in polyps. They proposed the nebulized budesonide as a relatively safe remedy for managing patients with SNP. The results of our study were compatible with Wang's results. Of course, the current study was conducted over six months after FESS, but the patients in Wang's study had not undergone FESS, and it was only a 14-day clinical trial study. Also, in a study conducted by Kang et al. (12), the effect of nasal budesonide douching was investigated in patients with sino-nasal polyposis. The results of their study suggested that budesonide douching plays a positive role in preventing the recurrence of SNP, too.

In the present study, we used the Lund-Mackay Scoring and Lund-Kennedy Scoring system to compare the patients before and after the FESS. Using the Lund-Kennedy Scoring system has a great economic benefit because it imposes a very small cost on the treatment system and prevents the patient from exposure to additional radiation by CT scan. One of the strengths of the current study is the absence of significant differences between the three under-study groups considering patients’ age, sex, underlying Sampter's Triad, history of smoking, the extent of the disease according to Lund Mackay scoring system, and the extent of surgery. It means that the patients were homogenous before the start of the study. Another strong point of the present study was the homogeneity of the patients in the three groups regarding the surgical process and post-operative follow-up. In this way, the same surgeon operated on all patients, and the operating room conditions were the same. Post-operative follow-ups were also done by a specialist and his assistant, which made the conditions similar for everyone and reduced possible biases in the results. The present study had limitations, including the need for molecular and histopathological investigations. Many studies have investigated molecular factors such as interleukin 5, interleukin 10, TGF-B, etc., in polyposis. Some other studies consider the different histopathologic features of polyps (eosinophilic and inflammatory polyps). Conducting a study with a larger sample size and more detailed investigations considering the phenotypes and endotypes of polyposis is necessary. 

## Conclusions

 Budesonide nebulizing suspension, compared to betamethasone nasal drops, showed better effects on preventing the recurrence of SNP after FESS. 
